# The complete chloroplast genome of *Foonchewia coriacea* (Rubioideae: Rubiaceae): a monotypic species endemic to Guangdong, China

**DOI:** 10.1080/23802359.2020.1852903

**Published:** 2021-01-16

**Authors:** Ying Zhang, Sheng Chen, Xianyi Xu, Ruijiang Wang

**Affiliations:** a Key Laboratory of Plant Resources Conservation and Sustainable Utilization, South China Botanical Garden, Chinese Academy of Sciences, Guangzhou, PR China; b College of Life Sciences, University of Chinese Academy of Sciences, Beijing, PR China; c College of Life Sciences, South China Agricultural University, Guangzhou, PR China

**Keywords:** *Foonchewia*, plastome, Rubiaceae, phylogeny

## Abstract

*Foonchewia coriacea*, a monotypic genus of the Rubiaceae, is endemic to China. Its complete chloroplast genome was determined to be 153,114 bp in length and the GC content was 37.90%. The sequence includes a large single-copy region of 83,978 bp, a small single-copy region of 18,290 bp, and the inverted region is 25,423 bp in length. It contains 129 genes, including 84 protein-coding genes, 37 tRNA genes, and 8 rRNA genes. The maximum likelihood (ML) and Bayesian inference (BI) analyses revealed *F. coriacea* was closely related to *Dunnia sinensis* with strong bootstrap values, belonging to the subfamily Rubioideae.

*Foonchewia coriacea* (Dunn) Z. Q. Song, a monotypic genus of Rubiaceae, is an endemic species distributed in the eastern of Guangdong Province and Fujian Province, China (Wen and Wang 2012; Song and Xu 2016). It is characterized by subshrub habit with a height of 0.5–3 m, growing under the secondary mixed forests at altitudes of 430–1100 m, alongside small trees. Although its phylogenetic relationship with four chloroplast regions (*atpB-rbcL*, *rps16*, *ndhF,* and *rbcL*) was conducted previously (Wen and Wang 2012), no complete genome data had been reported until now. Therefore, we reported the complete chloroplast (cp) genome of *F. coriacea* (GenBank Accession Number: MT942688) to provide useful genomic information for evolutionary dynamics and conservation evaluation.

The fresh and healthy leaves of *F. coriacea* were collected from Chaozhou City, Mt. Fenghuangshan, Guangdong Province of China (23°54′32.96′′ N, 116°36′35.15′′ E, 1103 m). The voucher specimen (*Ruijiang Wang*, *Guobin Jiang*, *and Mingdeng Yuan 5858*) was deposited in the South China Botanical Garden Herbarium, Chinese Academy of Sciences (IBSC_0857717). The total genomic DNA was extracted from the silica-gel dried leaves following the modified CTAB method (Doyle and Doyle 1987). The quality and concentration of extracted DNA were evaluated using NanoDrop 2000 spectrophotometer (Thermo Fisher Scientific, Waltham, MA) and Qubit version 3.0 fluorimeter (Life Technologies, Carlsbad, CA).

Then the genomic library (paired-end, PE = 150 bp) was sequenced on the Illumina Hiseq X Ten platform at Beijing Genomics Institute (BGI) (Wuhan, China). Totally 2 Gb sequence reads were obtained and used to assemble the cp genome after filtering and trimming the low-quality reads and adaptor sequences. The complete cp genome was assembled by NOVOPlasty version 2.6.3 (Dierckxsens et al. 2017) using *Galium mollugo* (NC_036970) as the reference genome with k-mer of 39–59. The assembled genome was annotated using plastid genome annotator (Qu et al. 2019) (PGA, https://github.com/quxiaojian/PGA) and tRNA genes were annotated on ARAGORN (Laslett and Canback 2004). In order to ensure the accuracy, the raw annotations were manually examined and adjusted in Geneious version 11.0.3 (Kearse et al. 2012).

The complete cp genome of *F. coriacea* was 153,114 bp with the typical quadripartite structure of angiosperms, including a large single-copy region (LSC) of 83,978 bp, a small single-copy region (SSC) of 18,290 bp, and the inverted repeat region (IR) of 25,423 bp. The genome harbored 129 genes, including 37 tRNA genes, 8 rRNA genes, and 84 protein-coding genes. The overall GC content in the cp genome of *F. coriacea* was 37.90%, which the corresponding value of the LSC, SSC, and IR regions were 35.80%, 32.20%, and 43.50%, respectively.

The phylogenetic position of *F. coriacea* was inferred with an additional 19 cp genomes from Rubiaceae family (Wikström et al. 2020; Zhang et al. 2020), using *Buddleja colvilei* Hook. f. & Thoms (Loganiaceae) (NC_042766) as outgroup. A maximum likelihood (ML) tree was constructed by using IQ-TREE (Nguyen et al. 2015) with 1000 bootstrap replicates under the TVM + F+R3 substitution model. Bayesian inference (BI) was also performed with MrBayes version 3.2.6 (Ronquist et al. 2012). The ML and BI trees were consistent and robust, showing *F. coriacea* had a close relationship with *Dunnia sinensis*, which formed an independent clade of the subfamily Rubioideae (Figure 1). The phylogenetic relationship of *F. coriacea* with complete genomic data was uncovered for the first time, largely enriching genetic resources for resolving the complex phylogeny relationship of Rubiaceae.

**Figure 1.**
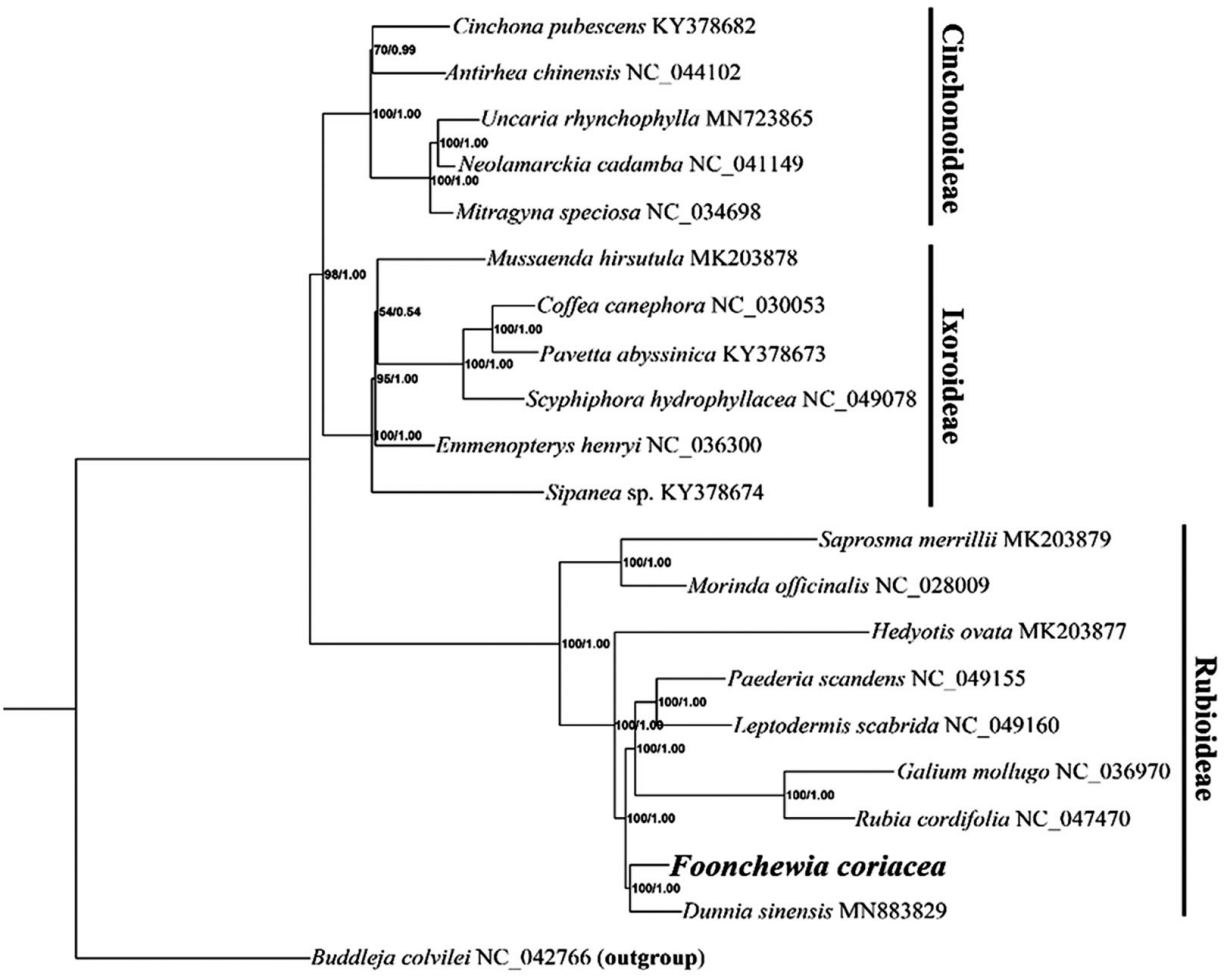
Phylogenetic relationships inference of the 20 (including *F. coriacea*) whole cp genome sequences in Rubiaceae based on ML and BI methods. Bootstrap values (left)/Bayesian posterior probability (right) were marked on the branches.

## Data Availability

The data that support the findings of this study are openly available from the corresponding author, or in NCBI (https://www.ncbi.nlm.nih.gov/) with the raw sequencing data (SRR12917150) and the accession number of MT942688.
